# Green Tea Extract Containing *Piper retrofractum* Fruit Ameliorates DSS-Induced Colitis via Modulating MicroRNA-21 Expression and NF-κB Activity

**DOI:** 10.3390/nu14132684

**Published:** 2022-06-28

**Authors:** Mak-Soon Lee, Jumi Lee, Yangha Kim

**Affiliations:** 1 Department of Nutritional Science and Food Management, Ewha Womans University, Seoul 03760, Korea; troph@hanmail.net (M.-S.L.); epiecess@naver.com (J.L.); 2 Graduate Program in System Health Science and Engineering, Ewha Womans University, Seoul 03760, Korea

**Keywords:** green tea, *Piper retrofractum*, colitis, microRNA, NF-κB, inflammatory response

## Abstract

The aim of the present study was to examine the effect of green tea extract containing *Piper retrofractum* fruit (GTP) on dextran-sulfate-sodium (DSS)-induced colitis, the regulatory mechanisms of microRNA (miR)-21, and the nuclear factor-κB (NF-κB) pathway. Different doses of GTP (50, 100, and 200 mg/kg) were administered orally once daily for 14 days, followed by GTP with 3% DSS for 7 days. Compared with the DSS-treated control, GTP administration alleviated clinical symptoms, including the disease activity index (DAI), colon shortening, and the degree of histological damage. Moreover, GTP suppressed miR-21 expression and NF-κB activity in colon tissue of DSS-induced colitis mice. The mRNA levels of inflammatory mediators, such as tumor necrosis factor-alpha (TNF-α), interleukin 6 (IL-6), interleukin-1β (IL-1β), inducible nitric oxide synthase (iNOS), and cyclooxygenase-2 (COX-2), were downregulated by GTP. Colonic nitric oxide (NO) and prostaglandin E2 (PGE2) production, and myeloperoxidase (MPO) activity were also lowered by GTP. Taken together, our results revealed that GTP inhibits DSS-induced colonic inflammation by suppressing miR-21 expression and NF-κB activity, suggesting that it may be used as a potential functional material for improving colitis.

## 1. Introduction

Colitis is a disease that causes inflammation in the colon and includes inflammatory bowel disease (IBD) and irritable bowel syndrome [1]. IBD is characterized by chronic relapsing inflammation affecting the digestive system and is classified into two major forms—ulcerative colitis (UC) and Crohn’s disease (CD) [2]. The pathogenesis of IBD has not yet been fully identified, but environmental, genetic, and immunological factors are known to interact to produce the disease [3]. UC is an inflammatory disease that occurs in the mucosal and submucosal layers of the large intestine (colon and rectum) [4]. In particular, UC patients have a low quality of life with clinical symptoms such as chronic diarrhea, bloody stool, abdominal pain, and weight loss, and a high risk of developing colorectal cancer [5].

MicroRNAs (miRs) are small endogenous non-coding RNA about 18–22 nucleotide molecules in length, which regulate gene expression by binding to the complementary sequence of a target mRNA. miRs are directly or indirectly involved in various functions, such as cell differentiation, proliferation, apoptosis, inflammation, metabolism, and oncogenesis [6,7]. miR-21 is upregulated in intestinal inflammation and tissue damage, whereas loss of miR-21 may prevent the development or progression of IBD [8]. In a study with IBD patients, miR-21 and miR-126 showed increased levels in UC, and miR-21 showed significantly higher levels in UC compared with CD. It was reported that miR-21 could act as a potential marker to discriminate between UC and CD [9]. Therefore, miR-21 has the potential to be used as a target biomarker for the treatment of colitis-related diseases [10,11].

The nuclear factor-kappa B (NF-ĸB)-mediated signaling pathway plays a central role among several signaling systems that cause inflammatory responses in inflammatory diseases [12]. NF-κB is a transcription factor that regulates the expression of specific genes in the nucleus, and its activation promotes the expression of over 150 target genes [13]. The activation of NF-κB induces an increase in various pro-inflammatory mediators, such as tumor necrosis factor-alpha (TNF-α), interleukin 6 (IL-6), interleukin-1β (IL-1β), inducible nitric oxide synthase (iNOS), and cyclooxygenase-2 (COX-2) [12]. The upregulation of iNOS and COX-2 increases the production of nitric oxide (NO) and prostaglandin E2 (PGE2) [14]. Therefore, inhibition of NF-κB activity has a potentially therapeutic role in regulating the inflammatory response.

Green tea (*Camellia sinesis* L.) is one of the oldest and most consumed tea beverages in the world. Green tea polyphenols are composed of catechins and their derivatives. Some of the major catechins in tea are: (−)-epigallocatechin-3-gallate (EGCG), (−)-epicatechin-3-gallate (ECG), (−)-epigallocatechin (EGC), and (−)-epicatechin (EC). Green tea catechins have various physiologically active functions, including antioxidant and anti-inflammatory effects, as well as beneficial health effects [15]. In a mouse model of colitis, it has been reported that green tea polyphenols have anti-inflammatory activity similar to sulfasalazine, a drug used to treat UC and CD [16].

Piperine is a natural alkaloid, the pungent compound found in spices, such as *Piper nigrum* (black pepper) and *Piper retrofractum* (Javanese long pepper) [17]. *Piper retrofractum*, belonging to the family Piperaceae, is one of the *Piper* species and is indigenously grown in Indonesia [18]. Piperine has been reported to reduce or prevent colonic inflammation in dextran sulfate sodium (DSS)-induced IBD [19]. Especially, EGCG has been reported to increase the bioavailability of piperine and significantly enhance the antioxidant effects of colitis when co-administered with piperine [20,21]. Therefore, we hypothesized that a mixture of green tea extract and *P. retrofractum* fruit containing piperine could reduce colonic inflammation. In this study, we investigated whether green tea extract containing *P. retrofractum* fruit (GTP) had beneficial effects on DSS-induced colitis. In addition, to elucidate the regulatory mechanism of GTP in colonic inflammation, we analyzed the pro-inflammatory mediators, including the miR-21 and NF-κB pathways.

## 2. Materials and Methods

### 2.1. GTP Preparation

GTP was kindly supplied by Newtree (Seoul, Korea). Green tea extract powder was purchased from Naturex (Avignon, France). *Piper retrofractum* fruit was extracted with 70% ethanol at 60 °C for 8 h. The extract of *P. retrofractum* fruit was mixed with the green tea extract powder at a ratio of 1:99 (*w*/*w*). The mixtures were concentrated and sterilized at 90–95 °C for 15–30 min, then spray dried to a powder. The levels of catechins and piperine in GTP were analyzed by high-performance liquid chromatography (HPLC) analysis using a Nanospace SI-2 HPLC system (Shiseido Co., Tokyo, Japan) equipped with a Capcell Pak C18 UG 120 column (4.6 × 250 mm, 5 µm; Shiseido, Tokyo, Japan). For the analysis of catechins, gradient elution was performed as follows, using 0.1% phosphoric acid in water (solvent A) and 0.1% acetic acid in acetonitrile (solvent B) as the mobile phase: 0–2 min 10% B, 3–10 min 15–20% B, 12–16 min 90% B, 17–20 min 10% B; flow rate of 1 mL/min, 35 °C. The detector wavelength was 280 nm, and the injection volume was 20 μL. For the analysis of piperine, gradient elution was performed as follows, using water (solvent A) and methanol (solvent B) as the mobile phase: 0–20 min 80% B; flow rate of 1 mL/min, 40 °C. The detector wavelength was 343 nm, and the injection volume was 10 μL.

### 2.2. Animals

Six-week-old male C57BL/6 mice (20–22 g; Doo Yeol Biotech Co., Seoul, Korea) were maintained on a 12/12 h light/dark cycle at 25 ± 1 °C and 55% humidity. All mice were individually housed in a plastic cage, and chow diet (2018S Teklad rodent diet; Envigo, Indianapolis, IN, USA) and water were available *ad libitum* throughout the study. The total sample size of mice was 35, divided into 7 per group. At the end of the experiment, the mice were fasted overnight and anesthetized with tiletamine/zolazepam/xylazine. Blood samples were collected by cardiac puncture through a central longitudinal incision in the abdominal wall. The animal experiments were performed according to the National Institutes of Health (NIH) international guidelines. The protocol was approved by the Institutional Animal Care and Use Committee (IACUC) of Ewha Womans University (IACUC No. 20-101).

### 2.3. Experimental Design

**Figure 1:**
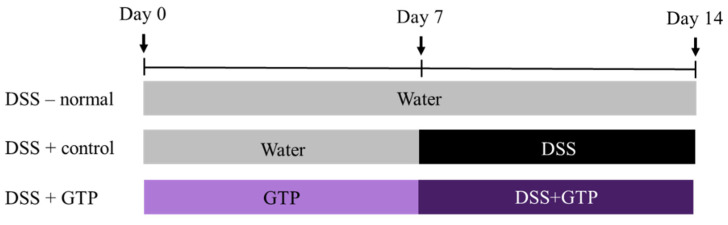
Experimental design for the administration of green tea extract containing *Piper retrofractum* (GTP) and dextran sulfate sodium (DSS).

Colitis was induced with 3% (*w*/*v*) DSS (molecular mass 36,000–50,000; MP Biomedicals, Santa Ana, CA, USA) dissolved in distilled water. The mice were randomly divided into five groups (*n* = 7 per group): DSS^−^ (water only; DSS-untreated normal), DSS^+^ (DSS only, DSS-treated control), GTP50 (DSS + 50 mg/kg GTP), GTP100 (DSS + 100 mg/kg GTP), and GTP200 (DSS + 200 mg/kg GTP). The DSS^−^ normal group was given water for 14 days; the DSS^+^ control group was given water for the first 7 days and then 3% DSS for the following 7 days; the GTP groups (50, 100, and 200 mg/kg, respectively) were given GTP orally by gavage for 14 days and were simultaneously given the water containing 3% DSS starting at Day 8 (Figure 1).

### 2.4. Clinical Evaluation

To evaluate the protective effect of GTP in DSS-induced colitis, mice were pre-treated with GTP for 7 days (Day 1–7), and fed 3% DSS-containing water ad libitum for another 7 days (Day 8–14). We evaluated clinical symptoms, such as DAI (weight loss, stool consistency, and rectal bleeding), colon shortening, and histological analysis. The disease activity index (DAI) was evaluated daily after DSS treatment and scored (Table 1). DAI was calculated as the total score (weight loss, stool consistency, and rectal bleeding) divided by 3 [22]. Resected distal colon tissues were fixed immediately in 10% formalin for histological evaluation. Paraffin-embedded tissue samples were serially sectioned, and one section from each mouse was stained with hematoxylin and eosin (H&E). Stained colon sections were observed under an Olympus microscope (Tokyo, Japan) at 200× magnification. Histological scores were measured as previously described [23].

### 2.5. Aspartate Aminotransferase (AST) and Alanine Aminotransaminase (ALT) Assay

Blood samples were centrifuged at 1500× *g* for 20 min at 4 °C. Serum AST and ALT levels were measured using a commercial enzymatic assay kit (Embiel, Gunpo, Korea) according to the manufacturer’s protocol.

### 2.6. Enzyme-Linked Immunosorbent Assay (ELISA)

The NF-κB activity was measured using the Phospo-NF-κB p65 ELISA kit (RayBiotech, Inc., Norcross, GA, USA) following extraction of the nuclear protein from colon tissue using a Nuclear Extraction Kit (Abcam, Cambridge, UK). Myeloperoxidase (MPO) levels were determined in the colon tissue homogenates using a Mouse MPO ELISA kit (Abcam, Cambridge, UK). PGE2 concentration was determined using the Prostaglandin E2 (PG-E2) ELISA kit (Cusabio, Wuhan, China) according to the manufacturer’s protocol. Absorbance was determined using a microplate reader (Varioskan Flash, Thermo Fisher Scientific, Waltham, MA, USA). Results were normalized to total protein concentration, determined by the bicinchoninic acid (BCA) assay (BCA Protein Assay Kit, Thermo Fisher Scientific, Pittsburgh, PA, USA).

### 2.7. NO Determination

NO level in colon tissue was measured as nitrite using a Griess reagent kit (Thermo Fisher Scientific, Pittsburgh) [24]. The nitrite concentration was determined using sodium nitrite as a standard. Results were normalized to total protein concentration measured by a BCA Protein Assay Kit (Thermo Fisher Scientific, Pittsburgh).

### 2.8. Real-Time Quantitative Polymerase Chain Reaction (RT-qPCR) 

The mRNA and miRNA levels were measured by RT-qPCR analysis, as previously described [25]. Total RNA from the colon was extracted using TRIzol reagent (GeneAll Biotechnology, Seoul, Korea). cDNA for mRNA and miRNA was synthesized from RNA extracted using M-MLV Reverse Transcriptase (Bioneer, Daejeon, Korea) and the miRNA cDNA Synthesis Kit with Poly (A) Polymerase Tailing (ABM, Inc., Richmond, BC, Canada). Afterward, RT-qPCR was performed in a Rotor-Gene Q thermocycler (Qiagen, Hilden, Germany) using GreenStar qPCR Master Mix (Bioneer). Primers used for RT-qPCR analysis are listed in Table 2. Target gene expression was normalized using β-actin as an endogenous control. The miR-21 and U6 specific primers were purchased from ABM Inc., and U6 snRNA was used as the reference control. The 2^−ΔΔ*C*^*^t^* method [26] was used as a relative quantification strategy.

### 2.9. Statistical Analysis

All data are expressed as mean ± standard error. Statistical analysis was performed using SPSS software (version 26; IBM Corp., Armonk, NY, USA). Differences among groups were analyzed using a one-way analysis of variance (ANOVA), followed by Tukey’s multiple comparison test. Statistical significance was set at *p* < 0.05.

## 3. Results

### 3.1. Contents of Green Tea Catechin and Piperine in GTP

**Figure 2:**
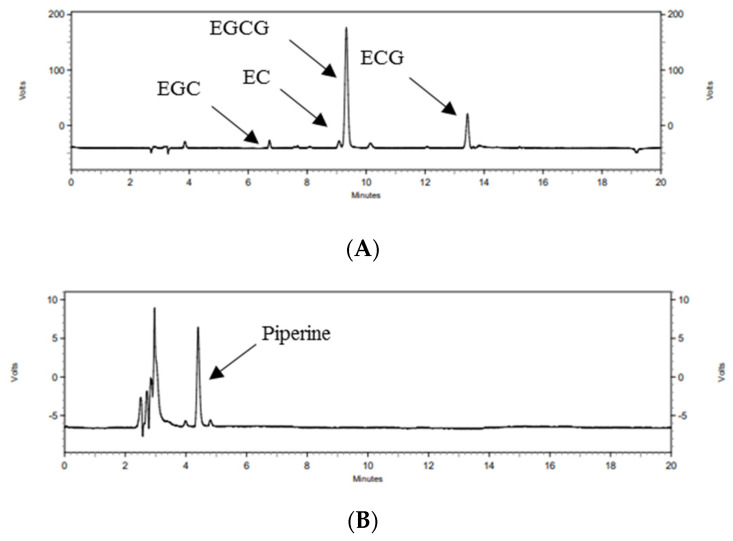
HPLC chromatogram of catechins (**A**) and piperine (**B**) in GTP. HPLC, high-performance liquid chromatography; EGC, epigallocatechin; EC, epicatechin; EGCG, epigallocatechin-3-gallate; ECG, epicatechin-3-gallate; GTP, green tea complex containing *Piper retrofractum* fruit.

Catechins and piperine in GTP were analyzed by HPLC (Figure 2), and the contents are shown in Table 3. The total catechin content including EGCG, ECG, EGC and EC in GTP was 791.99 ± 15.50 mg/g. The piperine content of GTP was 2.05 ± 0.13 mg/g.

### 3.2. Effect of GTP on AST and ALT Activities

**Figure 3:**
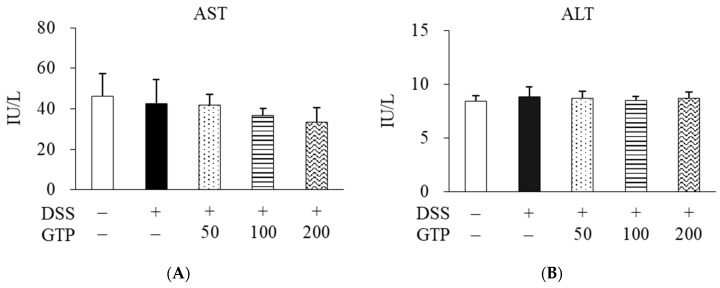
Effect of GTP on serum AST (**A**) and ALT (**B**) levels. Serum AST and ALT were measured by enzymatic colorimetric methods. Values are expressed as mean ± standard error (*n* = 7). GTP, green tea complex containing *Piper retrofractum* fruit; AST, aspartate aminotransferase; ALT, alanine aminotransaminase; DSS, dextran sulfate sodium.

Serum AST and ALT are enzymes present in liver cells, and their release into the blood increases when liver damage occurs. To investigate whether GTP induces liver damage, serum AST and ALT activities were measured in rats administrated GTP (50, 100, and 200 mg/kg). Serum AST and ALT activities were not significantly different among all groups (Figure 3).

### 3.3. GTP Ameliorated Clinical Symptoms in DSS-Induced Colitis Mice

**Figure 4:**
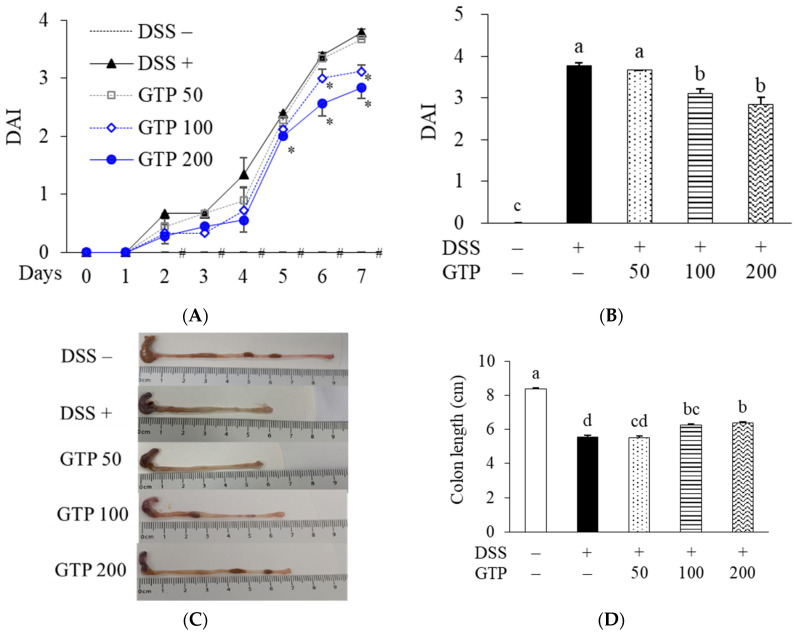

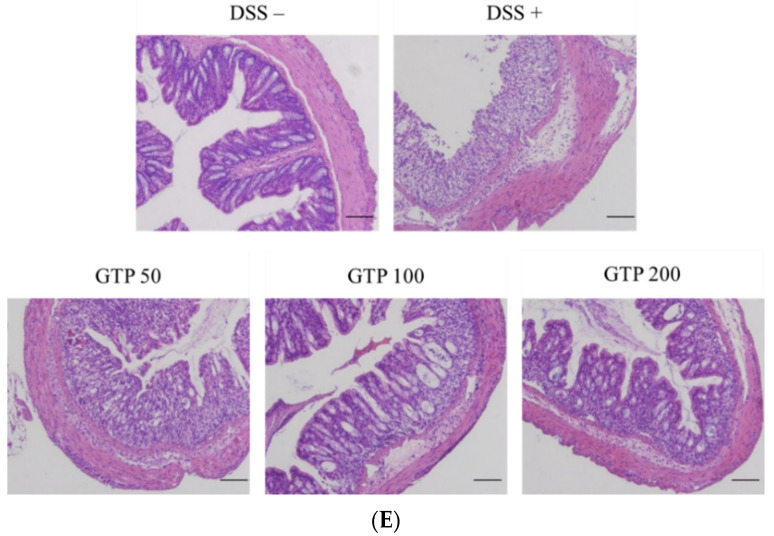

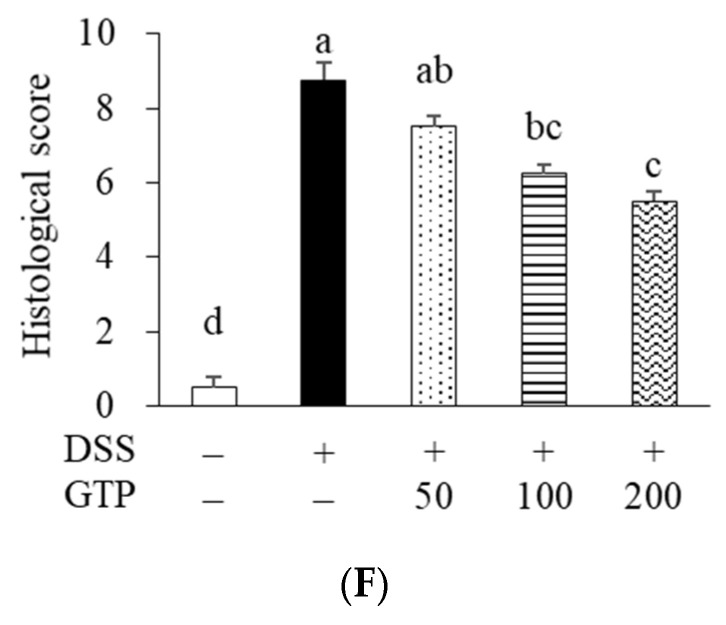
Effects of GTP on clinical symptoms in DSS-induced colitis in mice. (**A**,**B**) DAI score changes. (**C**) Colon morphology and (**D**) length. (**E**) Representative hematoxylin and eosin (H&E)-stained colon tissue (scale bars = 50 μm; magnification of 200×) and (**F**) the histological score. Values are expressed as mean ± standard error (*n* = 7). (**A**) Differences in mean values between the corresponding NOR and DSS groups were assessed by unpaired t-test; # *p* < 0.05. Differences in mean values among groups were assessed by using one-way ANOVA with Tukey’s multiple comparison tests * *p* < 0.05 versus DSS group. (**B**,**D**,**F**) Bars with different letters are significantly different at *p* < 0.05. GTP, green tea extract containing *Piper retrofractum* fruit; DSS, dextran sulfate sodium; DAI, disease activity index.

The DAI of the DSS-treated control group was elevated from Day 4 compared with the DSS-untreated normal group, and peaked on Day 7 (Figure 4A). Moreover, DSS induced colonic shortening (Figure 4C) and histological damage (Figure 4E). Administration of GTP (100 and 200 mg/kg) significantly ameliorated the DAI score (*p* < 0.05, Figure 4B), colonic shortening (*p* < 0.05, Figure 4D), and histological score (*p* < 0.05, Figure 4F) compared with the DSS-treated control group, but had no significant effect at the low dose (50 mg/kg).

### 3.4. GTP Suppressed Colonic MiR-21 Expression and NF-κB Activity

**Figure 5:**
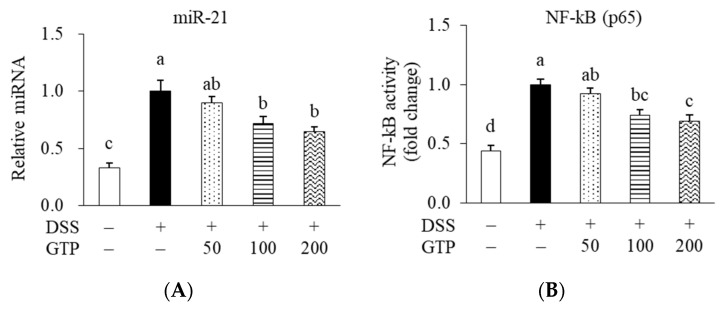
Effect of GTP on miR-21 expression (**A**) and NF-κB activity (**B**) in colon tissue. Values are expressed as mean ± standard error (*n* = 7). Bars with different letters are significantly different at *p* < 0.05. GTP, green tea extract containing *Piper retrofractum* fruit; miR-21, microRNA-21; NF-κB, nuclear factor-kappa B; DSS, dextran sulfate sodium.

DSS treatment increased miR-21 expression, whereas GTP (100 and 200 mg/kg) treatment suppressed miR-21 expression (by 28.3 and 35.2%, respectively) compared with the DSS-treated control group (*p* < 0.05, Figure 5A). In addition, the NF-κB activity in the GTP100 and GTP200 groups was inhibited by 25.4 and 30.6%, respectively, compared with the DSS-treated control group (*p* < 0.05, Figure 5B).

### 3.5. GTP Reduced Colonic Inflammation in DSS-Induced Colitis Mice

**Figure 6:**
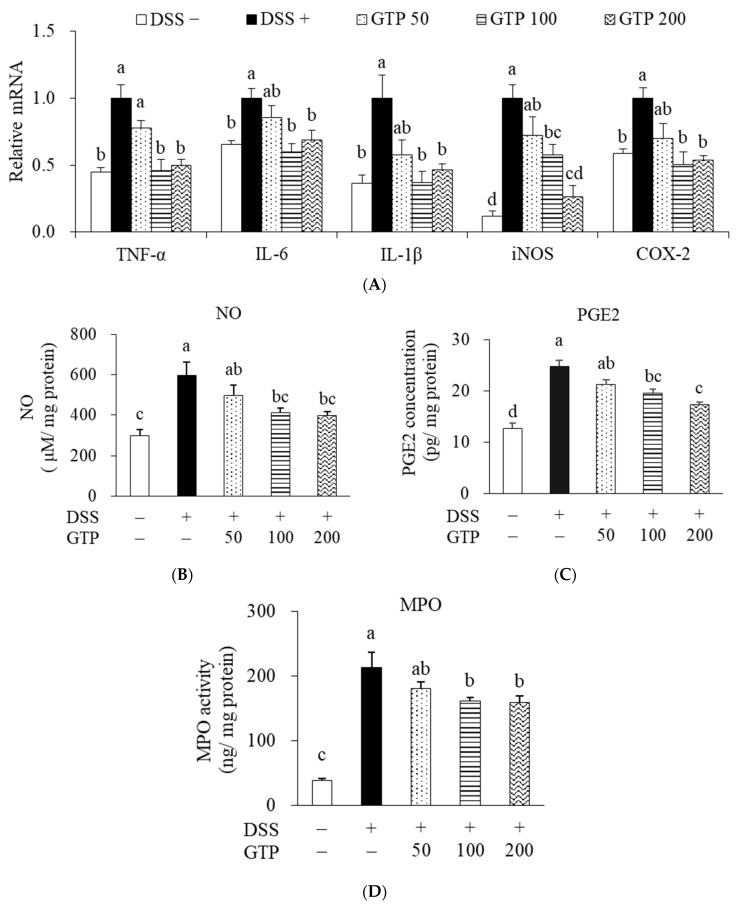
Effects of GTP on colonic inflammation. (**A**) The mRNA levels of TNF-α, IL-6, IL-1β, iNOS, and COX-2. The concentrations of NO (**B**) and PGE2 (**C**). (**D**) MPO activity. Values are expressed as mean ± standard error (*n* = 7). Bars with different letters are significantly different at *p* < 0.05. GTP, green tea extract containing *Piper retrofractum* fruit; TNF-α, tumor necrosis factor-alpha; IL-6, interleukin 6; IL-1β, interleukin-1β; iNOS, inducible nitric oxide synthase; COX-2, cyclooxygenase-2; NO, nitric oxide; PGE2, prostaglandin E2; DSS, dextran sulfate sodium.

DSS treatment significantly increased the expression levels of inflammatory mediators, such as TNF-α, IL-6, IL-1β, iNOS, and COX-2, compared to the DSS-untreated normal group (*p* < 0.05, Figure 6A). GTP treatment dose-responsively prevented an increase in the mRNA levels compared to the DSS-treated control group (Figure 6A). Based on our observation that iNOS and COX-2 expression promoted NO and PGE2 production, we investigated whether the inhibitory effects of GTP on the expression of iNOS and COX-2 are related to the inhibition of NO and PGE2 production. The concentrations of NO and PGE2 in colon tissue of the DSS-treated control group were significantly higher than those of the DSS-untreated normal group. The NO content in the GTP100 and GTP200 groups was reduced by 31.4 and 33.3%, respectively (*p* < 0.05, Figure 6B), and the PGE2 concentration was also inhibited by 21.2 and 30.2%, respectively, compared to the DSS-treated control group (*p* < 0.05, Figure 6C). The activity of MPO, an enzyme specific to neutrophil infiltration, was 5.6-fold higher in the DSS-treated control group compared to the DSS-untreated normal group, whereas MPO activity in the GTP100 and GTP200 groups was reduced by 24.6 and 25.3%, respectively, compared with the DSS-treated control group (*p* < 0.05, Figure 6D).

## 4. Discussion

The DSS-induced colitis animal model is widely used because its features, such as weight loss, bloody diarrhea, and colonic mucosal ulceration, are similar to those of human colitis [22]. In the present study, we investigated the effect of GTP on disease activity, colon shortening, and histologic features in DSS-induced colitis. Furthermore, we confirmed the mechanism by which GTP regulates NF-κB target pathways and miR-21 expression in the inflammatory response of colon tissue. The amounts of total catechins (EGCG, ECG, EGC, and EC) and piperine found in GTP were 792.00 ± 15.43 and 2.05 ± 0.13 mg/g, respectively. Serum AST and ALT levels of rats administrated GTP (50, 100, and 200 mg/kg) for 14 days did not differ among all groups, suggesting that GTP did not affect liver damage at all dosages. In a previous study, green tea polyphenols and EGCG ameliorated colonic damage and histological scores in DSS-induced colitis [16]. Piperine alleviated the clinical features of DSS-induced colitis, specifically weight loss, diarrhea, rectal bleeding, colon length, and colon mucosal damage [19]. In addition, the combination of EGCG and piperine improved body weight loss and clinical course in DSS-induced colitis [20]. Our results indicated that GTP improved the clinical symptoms (weight loss, diarrhea, gross bleeding, colon shortening, and histological score) of DSS-induced colitis, suggesting that it is beneficial for the improvement of colitis.

According to previous studies, miR plays an important regulatory role in the pathological process of IBD and colitis-related colorectal cancer [10,11]. In particular, it has been reported that miR-21, which targets NF-κB, plays an important role in the inflammatory process of colitis and IBD [8,9,27]. EGCG has been shown to reduce elevated miR-21 levels in the renal of cadmium-induced chronic renal injury rats [28]. A combination of piperine, curcumin, and taurine reduced circulating miR-21 levels in sera of hepatocellular carcinoma patients [29]. However, whether food extract affects the miR-21 expression in the colon tissue of DSS-induced colitis mice has not yet been elucidated. In this study, we first found an increase in the miR-21 expression in the colon tissue of DSS-induced colitis mice, whereas the miR-21 level was significantly decreased by GTP administration. Thus, it can be speculated that the ameliorating effect of GTP on colitis may be partially related to the suppression of miR-21 expression.

NF-κB is an important transcription factor regulating the inflammatory response and is involved in the *trans-*activation of many genes [13]. Activation of NF-κB stimulates the production of inflammatory cytokines (TNF-α, IL-1β, and IL-6) and induces iNOS and COX-2, which, in turn, promotes the production of NO and PGE2 [12,13]. In many previous studies, it was reported that green tea polyphenols had anti-inflammatory effects in the inflammatory process of IBD [16,30,31,32,33,34]. EGCG reduced the levels of TNF-α, IL-6, and NF-κB mRNA expression, NF-κB protein, and MPO activity in colon tissue of rats with trinitrobenzene sulfonic acid (TNBS)-induced colitis [31,32]. Similar to the above results, EGCG reduced the protein levels of inflammatory cytokines (TNF-α, IL-1β, and IL-6) and MPO activity in colon tissue of mice with DSS-induced colitis [16,33,34]. Recently, piperine has been shown to inhibit the levels of colonic NF-κB protein and the mRNA expression of pro-inflammatory mediators (TNF-α, IL-1β, IL-6, iNOS, and COX-2) in rats of TNBS-induced colitis [35]. The secretion of NO and TNF-α and the accumulation of MPO were also decreased by piperine in colon tissue of mice with acetic acid-induced colitis [36]. This indicates that green tea polyphenol EGCG and black pepper piperine can alleviate colitis by regulating the colonic inflammatory response. Consistent with previous studies, our results indicated that GTP inhibited the activity of NF-κB and MPO, the expression of TNF-α, IL-1β, IL-6, iNOS, and COX-2, and the production of NO and PGE2 in colon tissues of mice with DSS-induced colitis. Therefore, it is suggested that GTP may demonstrate a favorable effect on the inhibition of colonic inflammation.

**Figure 7:**
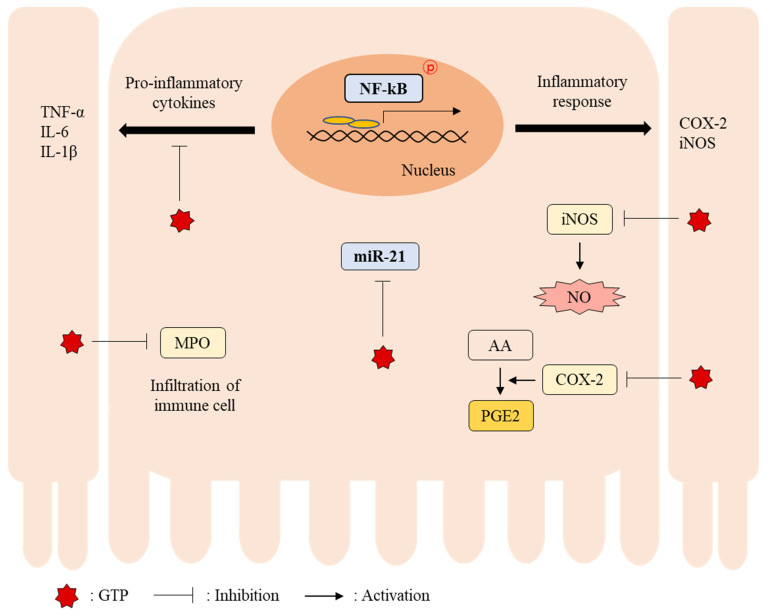
Schematic diagram of GTP-mediated regulatory mechanisms in the colon tissue of mice with DSS-induced colitis.; GTP, green tea extract containing *Piper retrofractum* fruit; DSS, dextran sulfate sodium; AA, arachidonic acid; COX-2, cyclooxygenase-2; IL-1β, interleukin-1β; IL-6, interleukin 6; iNOS, inducible nitric oxide synthase; MPO, myeloperoxidase; miR-21, microRNA-21; NF-κB, nuclear factor-kappa B; NO, nitric oxide; PGE2, prostaglandin E2; TNF-α, tumor necrosis factor-alpha.

In conclusion, our results suggest that GTP alleviates DSS-induced colitis by modulating miR-21 expression and NF-κB activity in the colon. Furthermore, it was partially associated with inhibited expression of colonic pro-inflammatory cytokine genes (TNF-α, IL-1β, and IL-6) as well as iNOS and COX-2, leading to decreased NO and PGE2 production and decreased MPO activity. (Figure 7). Thus, it suggests that GTP may be used as a potentially beneficial food material for the prevention/treatment of colitis. 

## Figures and Tables

**Table 1:** DAI scoring system for mice with DSS-induced colitis.

Score	Weight Loss (%)	Stool Consistency	Rectal Bleeding
0	None	Normal	Normal
1	1−5%		
2	5−10%	Loose	
3	10−20%		
4	>20%	Diarrhea	Gross bleeding

DAI: disease activity index; DSS, dextran sulfate sodium.

**Table 2:** Primer sequences used for qPCR.

Name	GenBank No.	Primer Sequence (5′–3′)
β-actin	NM_007393	F: GGACCTGACAGACTACCTCA
		R: GTTGCCAATAGTGATGACCT
COX-2	AF378830	F: ACAGTAACATCAAACCGACC
		R: GTGGAACCATTTCTAGGACA
IL-1β	M15131	F: TCCTCCTTGCCTCTGATGGG
		R: CATCCCCCACACGTTGACAG
IL-6	NM_031168	F: CCTTCCTACCCCAATTTCCA
		R: TAACGCACTAGGTTTGCCGA
iNOS	NM_001313922	F: CCACAGCAATATAGGCTCAT
		R: GGATTTCAGCCTCATGGTAA
TNF-α	NM_013693	F: AGCACAGAAAGCATGATCCG
		R: GCCACAAGCAGGAATGAGAA

β-actin, beta actin; COX-2, cyclooxygenase-2; IL-1β, interleukin-1 beta; IL-6, interleukin-6; iNOS, inducible nitric oxide synthase; 2; TNF-α, tumor necrosis factor-alpha.

**Table 3:** Contents of catechin and piperine in GTP.

Compound	Content (mg/g)
EGCG	522.04 ± 9.84
ECG	111.22 ± 2.06
EGC	105.67 ± 2.51
EC	53.06 ± 1.09
Piperine	2.05 ± 0.13

Data are expressed as mean ± standard error of six replicates. EGC, epigallocatechin; EC, epicatechin; EGCG, epigallocatechin-3-gallate; ECG, epicatechin-3-gallate; GTP, green tea complex containing *Piper retrofractum* fruit.

## Data Availability

The data presented in this study are available from the corresponding author upon request. The data are not publicly available due to privacy.
